# Human Gingival Fibroblast Attachment to Smooth Titanium Disks with Different Surface Roughnesses

**DOI:** 10.3390/biomimetics7040164

**Published:** 2022-10-14

**Authors:** Naoki Yanagisawa, Takayuki Ikeda, Masaki Takatsu, Kentaro Urata, Kensuke Nishio, Hideki Tanaka, Takayuki Kawato, Toshimitsu Iinuma

**Affiliations:** 1 Department of Complete Denture Prosthodontics, Nihon University School of Dentistry, 1-8-13 Kanda Surugadai, Chiyoda-ku, Tokyo 101-8310, Japan; 2 Department of Oral Health Sciences, Nihon University School of Dentistry, 1-8-13 Kanda Surugadai, Chiyoda-ku, Tokyo 101-8310, Japan

**Keywords:** peri-implantitis, titanium surface, surface roughness

## Abstract

Peri-implantitis is a significant problem associated with dental implants. It has been hypothesized that creating a soft-tissue seal around the implant neck prevents peri-implantitis. This study aims to clarify the effects of the surface smoothness of titanium disks on soft tissues. Thus, titanium disks were prepared through electrolytic composite polishing (ECP), sisal buffing (SB), hairline polishing (HP), and laser cutting (LC). The surface roughness values of seven items was measured. For ECP, SB, HP, and LC samples, the Ra values were 0.075, 0.217, 0.671, and 1.024 μm and the Sa values were 0.005, 0.115, 0.500, and 0.676, respectively, indicating that the surface roughness was remarkably lower with ECP. Moreover, the Wsk values for ECP, SB, HP, and LC were 0.521, 1.018, −0.678, and −0.558, respectively. The smooth surfaces produced by ECP and SB were biased toward the concave surface, whereas those produced by HP and LC were biased toward the convex surface. The Rku values for ECP, SB, HP, and LC were 2.984, 11.774, 14.182, and 26.232, respectively. Only the ECP exhibited a moderate bias peak and produced an extremely smooth surface. The contact angles in the cases of ECP, SB, HP, and LC were 60.1°, 66.3°, 68.4°, and 79.3°, respectively, indicating the hydrophobicity of the titanium disks. Human oral fibroblasts were then incubated on each disk for 24 and 48 h to measure cell attachment, and no significant differences were observed. The differences in Ra and Sa did not affect cell attachment. Therefore, by applying ECP to the abutment or implant neck, the cell attachment required for soft-tissue formation while preventing bacterial adhesion can be achieved.

## 1. Introduction

Implant treatment is a general term for treatments embedding devices in the body and is performed in the fields of dentistry and orthopedics. Dental implants are intended to restore the function of lost teeth; hence, they are generally used in a special environment, such as in the oral cavity containing many bacteria. Adequate bone mass and bone–implant integration are required for the long-term stability of dental implants [1,2,3,4]. Thus, significant research has been conducted to improve the implant surface on the bone [1,3,5,6,7,8,9,10,11,12]. However, peri-implantitis is a major problem in modern dental implants. The inflammation leads to bone resorption and implant failure, as well as morphological changes such as soft-tissue recession and marginal bone loss, which cause aesthetic problems. More than 1 × 10^9^ bacteria are present in the supragingival region of a single tooth, and approximately 1 × 10^3^ and 1 × 10^8^ bacteria are present in healthy shallow subgingival grooves and periodontal pockets, respectively [13]. Protecting the peri-implant tissue from these bacterial infections is key to preventing peri-implantitis [14,15,16,17].

Close contact and strong adhesion with soft tissue can create a seal around implant abutments and prosthetic components, preventing bacterial invasion [18]. Immediately after tooth extraction, the socket is filled with a thrombus and is completely replaced within 2–7 days with a gradual increase in the density of the granulation tissue [19]. Moreover, the initial epithelial healing after periodontal surgical interventions takes approximately 7–14 weeks, whereas the establishment of the biological width and barrier function around transmucosal implants or abutments takes 6–8 weeks [20]. For these reasons, the interaction between the connective tissue and titanium material is critical for successfully establishing a peri-implant seal. Therefore, in this study, we focused on the mucosal contact of the implant. Bacterial adhesion is positively correlated with surface roughness; therefore, the areas that may be exposed to saliva should be sanded and smoothed [21,22]. However, depending on the state of polishing, the surface of mechanically polished titanium can have some unevenness and undulations owing to the low thermal conductivity of titanium. The low thermal conductivity of titanium concentrates heat on the polishing tool, resulting in a poor polishing performance and difficulty in controlling the waviness on the titanium’s surface. 

The mucosal contact surface of the implant should adhere well to the soft tissue while preventing bacterial adhesion. This is a paradoxical situation that requires a smooth surface to prevent bacterial attachment and a rough surface to promote cell attachment. However, research on smooth surfaces in the field of implants has not progressed; thus, the effect of smoothness on cell attachment is not clear. In this study, the attachment of human fibroblasts to titanium surfaces prepared through laser cutting (LC), hairline polishing (HP), sisal buffing (SB), and electrolytic composite polishing (ECP) was examined to determine the effect of surface smoothness on soft tissue. 

## 2. Materials and Methods

### 2.1. Titanium Disks and Surface Treatment

Titanium disks (20 mm in diameter and 1.5 mm in thickness) were made from commercially pure grade 2 titanium (yield strength (0.2% offset): 39,900 psi; tensile strength: 49,900 psi; specifications met: ASTM B348 Gr2) (Standard Test Pieces, Kanagawa, Japan). The disks were produced by cutting a titanium cylinder with a diameter of 20 mm using a laser (TRUMPF Japan, Yokohama, Japan) with an output of 500 W and a frequency of 20,000 Hz.

The titanium disks were prepared with four types of polishing: ECP, SB, HP, and LC. ECP is a polishing technique that achieves electrochemical polishing via electropolishing and physical polishing (physically scraping the surface) with abrasives. SB was performed using an SB abrasive wheel (3M Japan Limited, Tokyo, Japan) with a rotation speed of 3000–3600/min. HP was performed in a single direction using a Scotch-Brite AVF320 scrub (3M Japan Limited, Shinagawa-ku, Tokyo, Japan) at a belt rotation speed of 11.7 m/s. The titanium disks with smooth surfaces were autoclaved and stored under dark ambient conditions for four weeks to standardize the properties of the titanium based on the documented time-dependent biological capability degradation of the titanium.

In total, 120 titanium disks were used in this study. Of these, 36 were used for surface characterization analyses, 36 for surface wettability measurements, and 48 for fibroblast attachment analyses.

### 2.2. Surface Morphology Characterization and Wettability of Titanium Disks

The surface roughness and morphology of the samples were measured at three random positions using a color 3D laser scanning microscope (VK-X3000, KEYENCE, Osaka, Japan). The surface roughness was measured using Ra, the arithmetic mean roughness; Rp, the maximum height; Rv, the maximum depth as the line roughness; Wsk, the skewness; Rku, the kurtosis; Sa, the three-dimensional arithmetic mean roughness; and Str, the aspect ratio of the surface texture as surface roughness. In addition, the hydrophobicity of the samples was evaluated by measuring the contact angle and the spread area of 10 μL droplets of double-distilled water (DD H_2_O). The contact angle was measured using a contact angle meter (CA-X, Kyowa Interface Science, Saitama, Japan) and the spread area was measured from the photographs of the droplets using ImageJ software (NIH, Bethesda, ML, USA).

### 2.3. Human Gingival Fibroblast Cell Culture

Human gingival fibroblast cells (human gingival fibroblast primary cell, ScienCell Research Laboratories, Carlsbad, CA, USA) were incubated at 37 °C in a humidified atmosphere containing 95% air and 5% CO_2_. The cells were grown in high-glucose (25 mM) Dulbecco’s modified Eagle medium (DMEM; Gibco-BRL, Rockville, MD, USA) supplemented with fetal bovine serum (FBS; Gibco-BRL, Rockville, MD, USA), fibroblast growth supplement (FGS; ScienCell Research Laboratories, Carlsbad, CA, USA), and antibiotics (penicillin/streptomycin; Sigma-Aldrich, St. Louis, MO, USA). After reaching 80% confluence, the cells were detached using a solution of 0.10% trypsin and 1 mM EDTA-4Na and seeded onto titanium disks placed in a 12-well culture dish at a density of 3 × 10^4^ cell/cm^2^. The culture medium (human oral epithelial cell culture complete growth media with serum and antibiotics; CELPROGEN, Torrance, CA, USA) was renewed every three days.

### 2.4. Cell Attachment and Density Assay

The attachment of the cells to the titanium disks was evaluated by measuring the number of cells on the surfaces of the titanium disks after incubation for 24 h using a colorimetric assay based on water-soluble tetrazolium salt (WST-8) (Cell Counting Kit-8; DOJINDO LABORATORIES, Kumamoto, Japan). The amount of formazan produced was measured at 420 nm using an enzyme-linked immunosorbent assay reader (SpectraMax ABS microplate-reader; MOLECULAR DEVICES, Tokyo, Japan). To evaluate the proliferative activity of the fibroblasts, the density of the propagated cells after 48 h was quantified using WST-8. The titanium disks were placed on a 12-well culture plate (inner diameter = 22.2 mm) and the cells were cultured for the prescribed time. After incubation, the titanium disks were gently transferred to a new 12-well culture plate for the WST-8 assay. Next, 100 µL of WST-8 was added to 1 mL of fresh medium containing the titanium disks. After culturing at 37 °C for 1 h, 100 μL of the culture solution was collected in 96-well plates and measured with a plate reader.

### 2.5. Statistical Analysis

The hydrophilicity of the smooth-surfaced titanium disks was evaluated using three different titanium disks (*n* = 9). Morphological experiments on the surface roughness of the titanium disks were performed using nine disks of each type (*n* = 9).

Cell attachment experiments were performed on six samples (*n* = 6). A one-way analysis of variance was performed to determine the differences between the smooth-surfaced titanium disk groups, and the statistical significance was set at *p* < 0.05. The Tukey multiple comparison test was used for the post hoc evaluation. A regression analysis was performed to determine the relationship between the surface roughness and cell attachment. Statistical analyses were performed using IBM SPSS Statistics version 20 (IBM, Armonk, NY, USA).

## 3. Results

### 3.1. Surface Morphology

**Figure 1:**
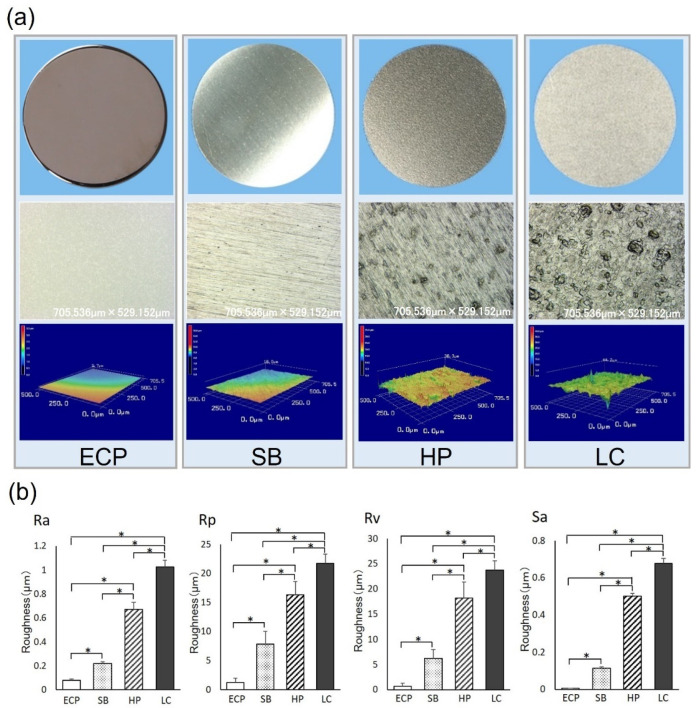
Properties of titanium surfaces with a smooth surface texture. (**a**) Top: Images of smooth titanium disks (20 mm diameter; ECP, SB, HP, LC). Middle: Scanning laser microscopy images. Bottom: The 3D images constructed from scanning laser microscopy images. (**b**) Histograms showing the surface roughness levels of Ra, Rp, Rv, and Sa. Data are presented as means ± SD (*n* = 9). Note: * *p* < 0.05; statistically significant differences between ECP, SB, HP, and LC surfaces.

The titanium disks subjected to different surface treatments had smooth surfaces. The disk subjected to ECP was glossy and had a mirror finish (Figure 1a, upper panel). In the scanning laser electron microscopic image, no unevenness was observed in the case of ECP. Lines were observed in the cases of SB and HP, and further depression was observed in the case of HP. Lines were not recognized in the case of LC; however, many depressions with large sizes were observed (Figure 1a, middle panel). The three-dimensional (3D) image clearly shows these features (Figure 1a, lower panel). The values of the surface roughness parameters (Ra, Rp, Rv, and Sa) were significantly lower in the case of ECP than those in the cases of SB, HP, and LC. The values of each parameter were in the following order: ECP > SB > HP > LC. Furthermore, the titanium disks in the case of HP exhibited significantly greater surface roughness than those in the cases of ECP and SB and significantly lower surface roughness than those in the case of LC. The titanium disks prepared by LC had significantly greater surface roughness than those in all other groups (Figure 1b). 

These results indicate that the titanium disks prepared by ECP had ultra-smooth surfaces compared to the surfaces of the titanium disks prepared through other polishing techniques. In the cases of ECP and SB, Wsk > 0; therefore, the waviness was biased downwards with respect to the average line. In contrast, in the cases of HP and LC, Wsk < 0; therefore, the waviness was biased upward with respect to the average line. Because the ECP satisfied the condition Rku < 3, the tips of fine projections on the surface were flat. Furthermore, the tips of the fine projections on the surfaces of SB, LC, and HP were sharp because these surfaces satisfied the condition Rku > 3. The disks in the cases of ECP and LC had isotropic surfaces due to Str being close to 1. Moreover, the disks in the case of SB had an anisotropic surface and those in the case of HP had intermediate properties (Table 1).

### 3.2. Wettability of Titanium Surface

**Figure 2:**
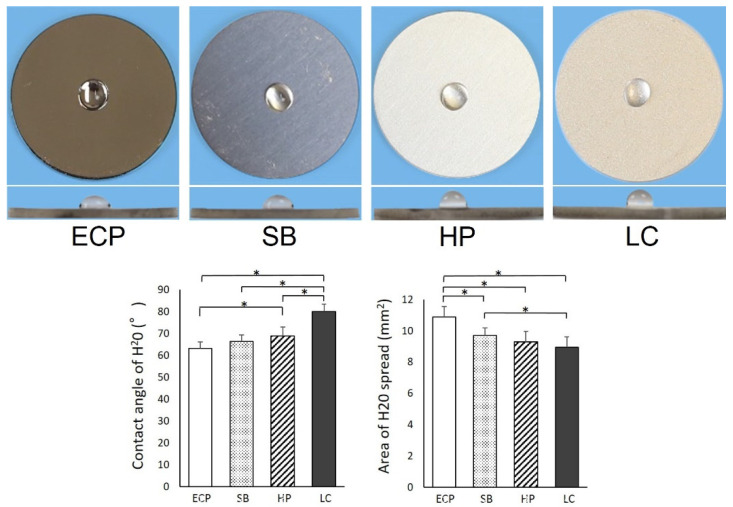
Wettability of each smooth surface. Image of a 10 μL DD H_2_O droplet pipetted onto a titanium surface (20 mm diameter). Histograms show the contact angle and area of 10 µL HO droplets. Data are presented as mean ± SD (*n* = 9). Note: * *p* < 0.05; statistically significant differences between ECP, SB, HP, and LC surfaces.

The wettability of the titanium surface is related to its surface physicochemical properties. The surfaces of the titanium disks of all groups were hydrophobic, and 10 µL of the DD H_2_O remained in the hemisphere without spreading. The water contact angle was 63° for the mildest ECP and 80° for the steepest LC. The contact angles and areas from LC were significantly different in all groups (Figure 2); moreover, the contact angles and areas from ECP were significantly different from those caused by HP and LC.

### 3.3. Human Gingival Fibroblast Attachment and Density

**Figure 3:**
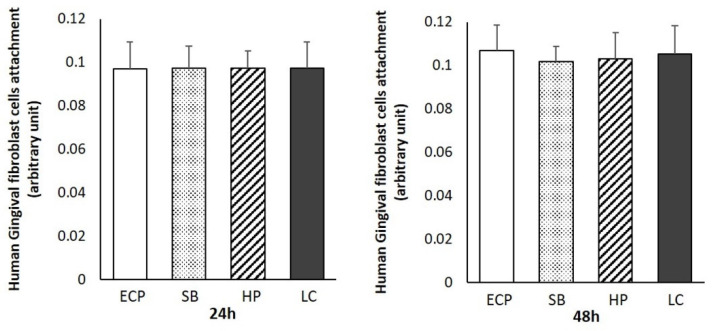
Total numbers of attached human gingival fibroblasts cultured for 24 h and density values after propagation for 48 h on titanium disks. Data are presented as means ± SD (*n* = 9).

The density values and total numbers of human gingival fibroblasts attached to the surfaces of the titanium disks were evaluated after incubation for 24 and 48 h (Figure 3). No differences were observed between the groups after 24 h of incubation. After 48 h, the numbers of human gingival fibroblasts attached to the titanium disks prepared by ECP and LC tended to be slightly higher than those in the other groups; however, the differences were not significant.

### 3.4. Relationship between Surface Roughness and Fibroblast Attachment and Density

**Figure 4:**
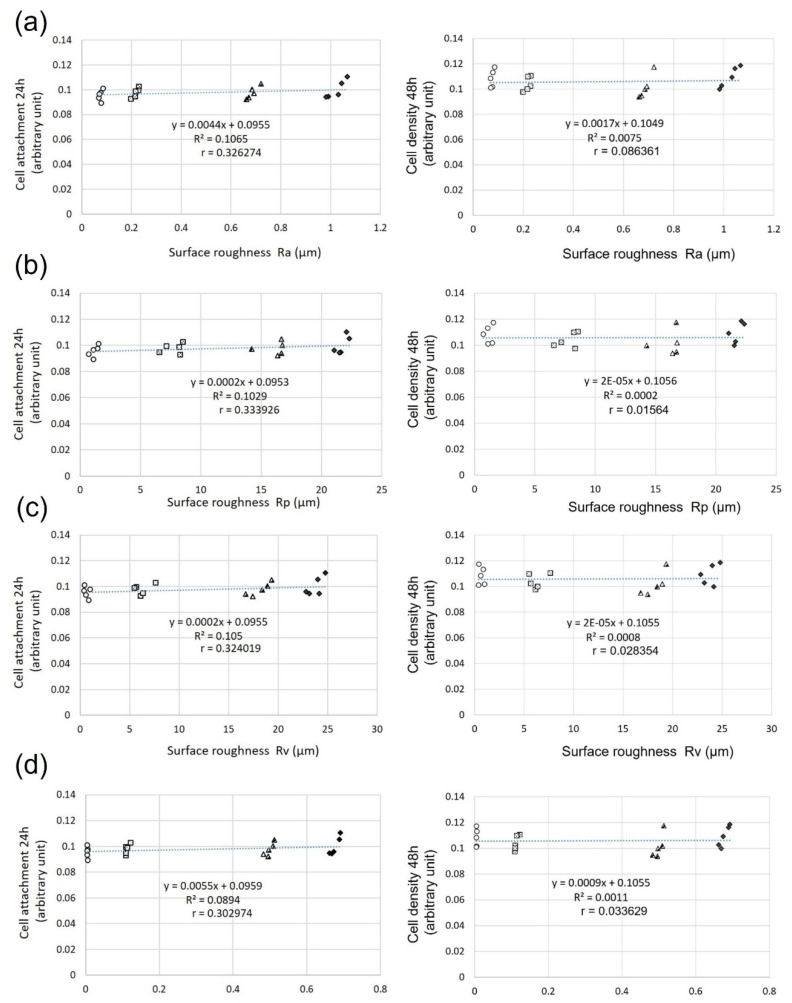
Relationship between cell attachment and surface roughness: (**a**) 24 h Ra (R = −0.326), 48 h Ra (R = 0.086); (**b**) 24 h Rp (R = −0.333), 48 h Rp (R = 0.015); (**c**) 24 h Rv (R = 0.324), 48 h Rv (R = 0.028); (**d**) 24 h Sa (R = 0.302), 48 h Sa (R = 0.033).

The relationship between the surface roughness (Ra, Rp, Rv, and Sa) and cell attachment was investigated using a regression analysis. For the regression analysis, we measured the Ra, Rp, Rv, and Sa values of the titanium disks used for the cell culture and the numbers of fibroblasts attached to them. Overall, a very weak positive correlation was observed between the surface roughness parameters and initial cell attachment after 24 h of incubation (Ra: R = −0.326, Rp: R = −0.333, Rv: R = −0.324, Sa = −0.302) (Figure 4, left panel). However, no correlation was observed after 48 h of culture (Ra: R = −0.863, Rp: R = −0.015, Rv: R = −0.028, and Sa = −0.033) (Figure 4, right panel).

## 4. Discussion

This study investigated smooth titanium surfaces in detail and clarified the relationship between the surface roughness of smooth surfaces and the adhesion of human fibroblasts. 

Research on the surface properties of titanium, which is an implant body, has thus far focused on improving the bone–implant integration (osseointegration); therefore, research on rough surfaces has been actively carried out. Studies on bone–implant integration range from those investigating the effect of carbon contamination on the titanium surface of the bone formation [1,23,24] to those showing enhanced osteoblast activity through the ultraviolet treatment of titanium [7,8,25,26,27,28]. However, most of these studies have used titanium, which has a relatively rough surface.

Peri-implantitis, an important problem in implant treatment, has been recently studied, and the smooth surface of titanium has attracted attention as a preventive and improvement measure for peri-implantitis. It has been reported that smooth surfaces are less susceptible to plaque and biofilm contamination than rough surfaces [29,30,31]. However, the effects of the different smoothness values are unknown. In contrast, areas exposed to plaque and biofilm contamination are covered with connective tissues such as the periodontal mucosa. The implant body is embedded in the bone and penetrates the connective tissue to connect with the abutment and superstructure. A connective tissue barrier is very important for protection against bacterial infections. Histological studies have reported that a fibroblast-rich barrier tissue around the implant surface plays an important role in maintaining the seal to the outside [32,33]. Therefore, the implant body located in the connective tissue barrier must be resistant to plaque and biofilm, and it should easily adhere to the fibroblasts.

The Sa classification is as follows: smooth (Sa < 0.5 μm), minimally rough (Sa = 0.5–1.0 μm), moderately rough (Sa = 1.0–2.0 μm), and rough (Sa > 2.0 μm) [34]. The prepared smooth surface is classified as smooth, except for the LC. However, under the Ra classification, it is considered the smoothest nanoroughness (Ra = 1–100 nm) [35]. These results for the surface roughness indicate that these four types differ from each other, even on the same smooth surface. Among them, ECP has the least unevenness, and the tops of the minute peaks are polished, indicating an isotropic ultra-smooth surface.

Titanium surfaces are chemically contaminated by the unavoidable deposition of hydrocarbons in a time-dependent manner, both in the laboratory and commercial use. Therefore, titanium surfaces lose hydrophilicity over time after surface treatment because of the accumulation of oxygen-containing hydrocarbons [1,23,36]. The surface roughness values of the four groups were compared, and the hydrophobicity was found to increase with an increase in surface roughness. Rough surface studies using osteoblasts have noted that hydrophilicity favors cell attachment. Although the titanium disk surfaces polished with ECP were more hydrophilic compared to those polished with LC, they did not exhibit complete hydrophilicity; therefore, the effect on cell attachment is localized [7,23,37,38,39].

Regardless of the culture time, the differences in cell attachment and density between the different groups were not significant. It has been reported that osteoblast attachment favors rough surfaces over smooth surfaces [3,6,40], and that the attachment of bacteria is positively correlated with the surface roughness [21,22]. This indicates that the Ra, Rp, Rv, and Sa in the 24 h cultures of human oral fibroblasts on smooth surfaces showed a very weak positive correlation (r = 0.3–0.5) with cell adhesion, while the surface roughness influenced the cell attachment. However, the correlation was very weak, with no significant differences in the overall cell adhesion. In addition, the correlation vanished in the 48 h cultures. Clinically, this was evidenced by the fact that the socket immediately after a tooth extraction was filled with a thrombus and the density of the granulation tissue gradually increased and were completely replaced within 2–7 days [19]. Moreover, in another study, fibroblasts were cultured on a surface whose surface energy was altered by plasma treatment; the cell adhesion after 20 min of incubation varied and disappeared over time [41]. The lack of difference in results after 24 h was similar to the results obtained herein, indicating that the short-term differences in cell adhesion are masked by the high proliferative capacity of fibroblasts. Considering the regeneration rate of granulation tissue, it is important that the cell adhesion and cell proliferation are not adversely affected after 24 h.

As mentioned above, the implant body located in the connective tissue barrier should have contradictory properties, i.e., being resistant to plaque and biofilm and prone to fibroblast attachment. Roughening the titanium surface can increase the fibroblast adhesion. Rough surfaces are an actively researched area; therefore, increasing the cell attachment is not difficult. However, in actual clinical practice, when the rough surface of an implant body is exposed in the oral cavity, it is covered with plaque and biofilm [29,30,31]. Peri-implantitis is then caused by bacteria within the biofilm. The microbiota associated with peri-implant diseases include *Peptostreptococcus* and *Staphylococci*. In addition, there are currently no standard treatment options. Therefore, the suppression of plaque and biofilm adhesion is an important countermeasure against peri-implantitis. Additionally, peri-implant soft tissue sealing and consequent protection are important [20,42,43,44]. This is because the seal around the implant can resist invasion by the above-mentioned bacteria. Therefore, it is noteworthy that the cell attachment is constant, regardless of the smoothness of the surface. In previous research on rough surfaces, the targets consisted of smooth surfaces obtained by mechanical polishing. Based on those results, the smoother the surface, the lower the cell attachment. However, the results of this study indicate that the cell attachment was almost constant on all smooth surfaces. This result is similar to the results obtained for fibroblasts in the rough surface studies with nanosized structures. This suggests that the nanosized surface roughness does not negatively affect the fibroblast attachment [45]. Moreover, the nanosized surface roughness did not affect the fibroblast adhesion, even on relatively smooth plasma-treated surfaces. The wettability levels of the smooth surfaces of ECP, SB, HP, and LC were significantly different. However, the contact angle and area were within the hydrophobic range (Figure 2). Numerous experiments have shown that hydrophilicity favors cell adhesion on rough surfaces. Rough surfaces, on the other hand, have been demonstrated to be more advantageous for cell adhesion than smooth surfaces. The difference in surface roughness between smooth and rough surfaces is considerably larger than the variations in surface roughness among smooth surfaces; therefore, the effect of surface roughness on cell attachment on smooth surfaces is considered to be small. This can be seen from the very weak positive correlation between cell adhesion and surface roughness after 24 h of culture (Figure 4). Therefore, the lack of difference in cell attachment may be due to the very weak effects of the wettability and surface roughness.

On the other hand, the implant surface roughness is closely correlated with the amount of attached bacteria. This is because rough surfaces provide a greater contact area between the surface and bacterial cells, providing protection from shear forces. An Ra value of 0.2 μm has been reported as a threshold for reducing bacterial adhesion [46,47,48,49]. In addition, it has been suggested that biofilms are also reduced on manually polished surfaces [49]. These results clearly indicate that the amounts of attached bacteria are positively related to the surface roughness. In clinical practice, one of the treatments for peri-implantitis is polishing the contaminated implant surface.

A very smooth surface, such as that obtained through ECP, may prevent plaque and biofilm attachment while fibroblasts adhere to the surroundings. The study of smooth surfaces has received minimal attention thus far and may be the subject of future research. However, a limitation of our study is that the cells reach high levels of confluence, thereby making it difficult to assess the differences between groups after long-term culture. Therefore, a long-term evaluation of the effects of smooth surfaces on connective tissue sealing is required. Finally, further in vivo studies are required to provide new strategies for promoting soft-tissue sealing and reducing the risk of peri-implantitis.

## 5. Conclusions

The smoothness of the titanium surface did not affect the amount of fibroblast attachment, despite the obvious difference in surface roughness. This connective tissue compatibility was considered constant within the smooth-classified surface roughness category. The use of a very smooth surface such as that obtained through ECP that addresses bacterial contamination is a promising strategy for improving connective tissue sealing and preventing bacterial entry into the peri-implant area.

## Figures and Tables

**Table 1:** Values for the skewness (Wsk), kurtosis (Rku), and aspect ratio of the surface texture (Str). Data are presented as means ± SD (*n* = 9).

	Wsk	Rku	Str
ECP	0.5213 ± 0.2252	2.984 ± 1.7168	0.8167 ± 0.0971
SB	1.0183 ± 0.8523	11.7790 ± 22.8187	0.0328 ± 0.0059
HP	−0.6783 ± 0.3770	14.1829 ± 3.1957	0.4243 ± 0.0847
LC	−0.5584 ± 0.2525	26.2324 ± 2.7717	0.8306 ± 0.0418

## Data Availability

The data presented in this study are available on request from the corresponding author.

## References

[1] Att W., Hori N., Takeuchi M., Ouyang J., Yang Y., Anpo M., Ogawa T. (2009). Time-dependent degradation of titanium osteoconductivity: An implication of biological aging of implant materials. Biomaterials.

[2] Kojima N., Ozawa S., Miyata Y., Hasegawa H., Tanaka Y., Ogawa T. (2008). High-throughput gene expression analysis in bone healing around titanium implants by DNA microarray. Clin. Oral Implants Res..

[3] Ogawa T., Nishimura I. (2003). Different bone integration profiles of turned and acid-etched implants associated with modulated expression of extracellular matrix genes. Int. J. Oral Maxillofac. Implants.

[4] Minamikawa H., Att W., Ikeda T., Hirota M., Ogawa T. (2016). Long-Term Progressive Degradation of the Biological Capability of Titanium. Materials.

[5] Lin Z., Rios H.F., Volk S.L., Sugai J.V., Jin Q., Giannobile W.V. (2011). Gene expression dynamics during bone healing and osseointegration. J. Periodontol..

[6] Ogawa T., Ozawa S., Shih J.H., Ryu K.H., Sukotjo C., Yang J.M., Nishimura I. (2000). Biomechanical evaluation of osseous implants having different surface topographies in rats. J. Dent. Res..

[7] Ueno T., Yamada M., Suzuki T., Minamikawa H., Sato N., Hori N., Takeuchi K., Hattori M., Ogawa T. (2010). Enhancement of bone-titanium integration profile with UV-photofunctionalized titanium in a gap healing model. Biomaterials.

[8] Att W., Hori N., Iwasa F., Yamada M., Ueno T., Ogawa T. (2009). The effect of UV-photofunctionalization on the time-related bioactivity of titanium and chromium-cobalt alloys. Biomaterials.

[9] Att W., Takeuchi M., Suzuki T., Kubo K., Anpo M., Ogawa T. (2009). Enhanced osteoblast function on ultraviolet light-treated zirconia. Biomaterials.

[10] Bereznai M. (2003). Surface modifications induced by ns and sub-ps excimer laser pulses on titanium implant material. Biomaterials.

[11] Gotz H.E., Muller M., Emmel A., Holzwarth U., Erben R.G., Stangl R. (2004). Effect of surface finish on the osseointegration of laser-treated titanium alloy implants. Biomaterials.

[12] Guilherme A.S., Henriques G.E., Zavanelli R.A., Mesquita M.F. (2005). Surface roughness and fatigue performance of commercially pure titanium and Ti-6Al-4V alloy after different polishing protocols. J. Prosthet. Dent..

[13] Slots J. (1979). Subgingival microflora and periodontal disease. J. Clin. Periodontol..

[14] Marín-Pareja N., Salvagni E., Guillem-Marti J., Aparicio C., Ginebra M.P. (2014). Collagen-functionalised titanium surfaces for biological sealing of dental implants: Effect of immobilisation process on fibroblasts response. Colloids Surf. B Biointerfaces.

[15] Gómez-Florit M., Xing R., Ramis J.M., Taxt-Lamolle S., Haugen H.J., Lyngstadaas S.P., Monjo M. (2014). Human gingival fibroblasts function is stimulated on machined hydrided titanium zirconium dental implants. J. Dent..

[16] Mombelli A., Décaillet F. (2011). The characteristics of biofilms in peri-implant disease. J. Clin. Periodontol..

[17] Mombelli A., Müller N., Cionca N. (2012). The epidemiology of peri-implantitis. Clin. Oral Implants Res..

[18] Squier C.A., Collins P. (1981). The relationship between soft tissue attachment, epithelial downgrowth and surface porosity. J. Periodontal. Res..

[19] Pippi R. (2017). Post-Surgical Clinical Monitoring of Soft Tissue Wound Healing in Periodontal and Implant Surgery. Int. J. Med. Sci..

[20] Clark E.A., Brugge J.S. (1995). Integrins and signal transduction pathways: The road taken. Science.

[21] Quirynen M., De Soete M., van Steenberghe D. (2002). Infectious risks for oral implants: A review of the literature. Clin. Oral Implants Res..

[22] Gatewood R.R., Cobb C.M., Killoy W.J. (1993). Microbial colonization on natural tooth structure compared with smooth and plasma-sprayed dental implant surfaces. Clin. Oral Implants Res..

[23] Aita H., Hori N., Takeuchi M., Suzuki T., Yamada M., Anpo M., Ogawa T. (2009). The effect of ultraviolet functionalization of titanium on integration with bone. Biomaterials.

[24] Lee J.H., Ogawa T. (2012). The biological aging of titanium implants. Implant Dent..

[25] Iwasa F., Hori N., Ueno T., Minamikawa H., Yamada M., Ogawa T. (2010). Enhancement of osteoblast adhesion to UV-photofunctionalized titanium via an electrostatic mechanism. Biomaterials.

[26] Ikeda T., Hagiwara Y., Hirota M., Tabuchi M., Yamada M., Sugita Y., Ogawa T. (2014). Effect of photofunctionalization on fluoride-treated nanofeatured titanium. J. Biomater. Appl..

[27] Tabuchi M., Ikeda T., Hirota M., Nakagawa K., Park W., Miyazawa K., Goto S., Ogawa T. (2015). Effect of UV Photofunctionalization on Biologic and Anchoring Capability of Orthodontic Miniscrews. Int. J. Oral Maxillofac. Implants.

[28] Ikeda T., Ueno T., Saruta J., Hirota M., Park W., Ogawa T. (2021). Ultraviolet Treatment of Titanium to Enhance Adhesion and Retention of Oral Mucosa Connective Tissue and Fibroblasts. Int. J. Mol. Sci..

[29] Amoroso P.F., Adams R.J., Waters M.G., Williams D.W. (2006). Titanium surface modification and its effect on the adherence of Porphyromonas gingivalis: An in vitro study. Clin. Oral Implants Res..

[30] Scheeren Brum R., Apaza-Bedoya K., Labes L.G., Volpato C.A.M., Pimenta A.L., Benfatti C.A.M. (2021). Early Biofilm Formation on Rough and Smooth Titanium Specimens: A Systematic Review of Clinical Studies. J. Oral Maxillofac. Res..

[31] Martines R.T., Sendyk W.R., Gromatzky A., Cury P.R. (2008). Sandblasted/acid-etched vs smooth-surface implants: Implant clinical reaction to experimentally induced peri-implantitis in Beagle dogs. J. Oral Implantol..

[32] Moon I.S., Berglundh T., Abrahamsson I., Linder E., Lindhe J. (1999). The barrier between the keratinized mucosa and the dental implant. An experimental study in the dog. J. Clin. Periodontol..

[33] Abrahamsson I., Berglundh T., Moon I.S., Lindhe J. (1999). Peri-implant tissues at submerged and non-submerged titanium implants. J. Clin. Periodontol..

[34] Albrektsson T., Wennerberg A. (2004). Oral implant surfaces: Part 1--review focusing on topographic and chemical properties of different surfaces and in vivo responses to them. Int. J. Prosthodont..

[35] Le Guéhennec L., Soueidan A., Layrolle P., Amouriq Y. (2007). Surface treatments of titanium dental implants for rapid osseointegration. Dent. Mater..

[36] Hori N., Att W., Ueno T., Sato N., Yamada M., Saruwatari L., Suzuki T., Ogawa T. (2009). Age-dependent degradation of the protein adsorption capacity of titanium. J. Dent. Res..

[37] Nakhaei K., Ishijima M., Ikeda T., Ghassemi A., Saruta J., Ogawa T. (2020). Ultraviolet Light Treatment of Titanium Enhances Attachment, Adhesion, and Retention of Human Oral Epithelial Cells via Decarbonization. Materials.

[38] Okubo T., Ikeda T., Saruta J., Tsukimura N., Hirota M., Ogawa T. (2020). Compromised Epithelial Cell Attachment after Polishing Titanium Surface and Its Restoration by UV Treatment. Materials.

[39] Ikeda T., Okubo T., Saruta J., Hirota M., Kitajima H., Yanagisawa N., Ogawa T. (2021). Osteoblast Attachment Compromised by High and Low Temperature of Titanium and Its Restoration by UV Photofunctionalization. Materials.

[40] Le Guehennec L., Lopez-Heredia M.A., Enkel B., Weiss P., Amouriq Y., Layrolle P. (2008). Osteoblastic cell behaviour on different titanium implant surfaces. Acta Biomater..

[41] Canullo L., Genova T., Gross Trujillo E., Pradies G., Petrillo S., Muzzi M., Carossa S., Mussano F. (2020). Fibroblast Interaction with Different Abutment Surfaces: In Vitro Study. Int. J. Mol. Sci..

[42] Weber H.P., Fiorellini J.P. (1992). The biology and morphology of the implant-tissue interface. Alpha Omegan.

[43] Buckley C.D., Rainger G.E., Bradfield P.F., Nash G.B., Simmons D.L. (1998). Cell adhesion: More than just glue (review). Mol. Membr. Biol..

[44] Figuero E., Graziani F., Sanz I., Herrera D., Sanz M. (2014). Management of peri-implant mucositis and peri-implantitis. Periodontol. 2000.

[45] Kubo K., Tsukimura N., Iwasa F., Ueno T., Saruwatari L., Aita H., Chiou W.A., Ogawa T. (2009). Cellular behavior on TiO2 nanonodular structures in a micro-to-nanoscale hierarchy model. Biomaterials.

[46] Bollen C.M., Lambrechts P., Quirynen M. (1997). Comparison of surface roughness of oral hard materials to the threshold surface roughness for bacterial plaque retention: A review of the literature. Dent. Mater..

[47] Teughels W., Van Assche N., Sliepen I., Quirynen M. (2006). Effect of material characteristics and/or surface topography on biofilm development. Clin. Oral Implants Res..

[48] Yoda I., Koseki H., Tomita M., Shida T., Horiuchi H., Sakoda H., Osaki M. (2014). Effect of surface roughness of biomaterials on Staphylococcus epidermidis adhesion. BMC Microbiol..

[49] McGaffey M., Zur Linden A., Bachynski N., Oblak M., James F., Weese J.S. (2019). Manual polishing of 3D printed metals produced by laser powder bed fusion reduces biofilm formation. PLoS ONE.

